# The Lead Extractor's Toolbox: A Review Of Current Endovascular Pacemaker And ICD Lead Extraction Techniques

**Published:** 2003-07-01

**Authors:** FA Bracke

**Affiliations:** Department of Cardiology, Catharina Hospital, Eindhoven, The Netherlands

## Introduction

Recently introduced pacemaker leads float freely within the veins and myocardium. Later on, fibrous encapsulation of the lead develops [1]. These adhesions not only occur at the lead tip but are commonly found anywhere along the whole length of the lead at sites where the lead is in contact with the vein or the myocardium [1-4].

These adhesions hamper lead removal as tight scar tissue can withhold the leads during traction. This not only occurs at the level of the flings and tines of passive fixation leads but at any level of the lead body, especially at sites of unequal diameter for example electrodes and defibrillator coils. Further the lead tip is often larger than the lead body due to the fixation mechanism and adhering scar tissue and can become impacted on withdrawal in the narrow canal provided by the fibrous envelope.

Force applied to leads is limited by the tensile strength of the insulation and conductor coils of the leads. They may severe with forceful traction, and denuded indwelling lead fragments have a higher incidence of thrombo-fibrotic complications and may maintain infection [5-8]. Force is also limited by the impact of traction on the veins and myocardium. Unopposed traction can lead to invagination of the myocardium, myocardial rupture, arrhythmia, hypotension or avulsion of a tricuspid valve leaflet [9-13].

Therefore, additional tools have been developed to assist in freeing the lead body from the adhesions as well as the lead tip from the myocardium, to prevent laceration of the myocardium and to provide enough room for the lead to be withdrawn whilst preventing disintegration of the lead. We describe the technical aspects of current endovascular techniques, the results, the complications and shortly discuss the indications.

## Technique of extraction

### Traction

Direct traction without additional tools is the most basic technique for lead extraction. Rosenheck et al. added 5- 10 times rotation of the lead with simultaneous gentle traction [14]. Another modification consists of prolonged graded traction [15-17]. For this purpose, increasing weights are connected to the proximal end of the lead. They are guided over a pulley mounted on the bed of the patient or, to keep the patient ambulatory, fixed under tension to the skin using rubber bands and adhesive tape.

### Locking stylets

To avoid disintegration of the lead during traction, a locking stylet is introduced into the central lumen of the lead. It consists of a straight non-expandable wire that can be locked into the coil close to the tip of the lead. The force exerted via a locking stylet is almost directly applied at the tip, bypassing most of the conductor and the insulation. Locking mechanisms differ between manufacturers and recently introduced devices can be unlocked and repositioned if necessary.

There are limitations to the use of a locking stylet. If the conductor is broken or distorted it is not possible to introduce the stylet. Excessive force can dislocate the stylet or the distal conductor coil can still unwind or even disconnect from the electrode. Similar as with direct traction it does not provide a solution for either the risk of invagination of the myocardium or for removing the bulbous tip through the fibrous sheaths along the body of the lead.

### Conventional intravascular counter traction

To overcome the limitations of a locking stylet, two telescoping synthetic sheaths can be alternately advanced over the lead. The fibrous bindings within the veins or myocardium can be mechanically disrupted, and at the same time enough room is created to remove the lead [18-19]. It is necessary to use a locking stylet: the leads are often to fragile to withstand the traction necessary to align the sheaths with the lead and to counter the forces applied to advance the sheath.

 Once the distal electrode is reached counter traction is applied: the larger bore outer sheath is positioned and held against the myocardium to prevent its inversion during traction on the locking stylet. The force is thus concentrated at a small area of the scar tissue without gross displacement of the myocardium.

 Although counter traction prevents invagination of the myocardium, perforation of the myocardium is still possible. The lead tip may have been incorporated into the myocardium, inevitably leading to a perforation after dislocation of the tip. Further, the possibility of increasing the force using counter-traction can lacerate the myocardium especially in the thin-walled atrium [20-21].

### Laser assisted extraction

**Figure 1:**
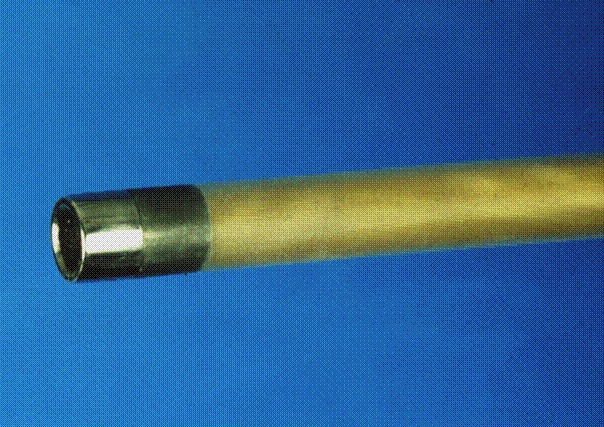
The Spectranetics laser sheath

**Figure 2:**
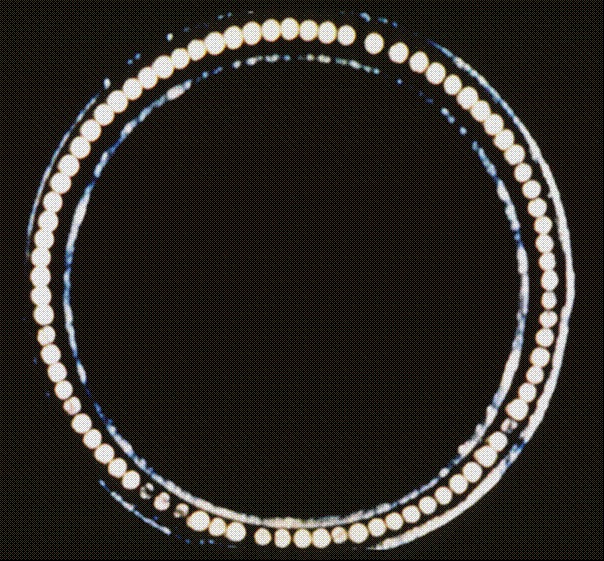
Tip of the laser sheath with the optical fibers circumferentially arranged around the tip

A laser sheath replaces the inner sheath with laser assisted lead extraction [22-25]. The laser sheath consists of optic fibers spirally warped between the inner and outer tubing of the sheath (Figure 1). At the tip of the device the fibers are arranged in a ring from which the pulsed laser light is emitted to ablate the tissue (Figure 2). Lasing energy is delivered with a 308 nm XeCl excimer laser (Spectranetics CVX-300) which emits pulsed light at a maximum fluence of 60 mJ/mm2 and a 40 - 80 Hz repetition rate. The ablation mechanism combines photochemical destruction of cellular structures with explosive photo-thermal vaporization of cellular water, which creates transient micro-bubbles to mechanically disrupt the tissue. As the penetration depth of 308 nm light in vascular tissue is approximately 100 micron, it is completely absorbed in the tissue immediately in front of the tip. This results in an ablation depth, depending on the applied force, between 2 and 15 microns per pulse in the experimental setting [26]. Force exerted on the sheath increases the mechanical effect of the micro-bubbles entrapped beneath the tip of the device in creating microscopic tears. The ablation results in a shearing of the fibrous scar, often leaving a rim around the lead. It has to be understood that the blunt tip of the laser sheath is not suited for direct mechanical disruption of the fibrous scar and that applying more force than necessary to assure good contact with the tissue does not improve efficacy but increases the risk of complications.

As with conventional sheaths the outer sheath helps to align the laser sheath with the lead and facilitates its handling by reducing friction with the surrounding tissue. Lasing can only be applied until the distal electrode to prevent myocardial perforation. Therefore, counter-traction is still necessary to dislocate the tip. The synthetic outer sheath is used for counter-traction as the inner diameter of the laser sheath is often too small to accommodate the lead tip and its attachments. It has to be noted that the laser has no effect on the insulation of the leads. To accommodate for different sizes of leads 12, 14 and 16 F sheaths are available.

### Electrosurgical sheaths

An emerging new technique is the bipolar electrosurgical dissection sheath. Two electrodes are mounted on the beveled tip of a synthetic sheath (similar to a conventional sheath) and radiofrequency energy is applied between them providing bipolar point dissection of scar tissue. Interestingly, directional dissection is theoretically possible by rotating the sheath to orientate the bipole. Similar as with a laser sheath, counter traction is used to dislocate the tip.

### Transfemoral approach

**Figure 3:**
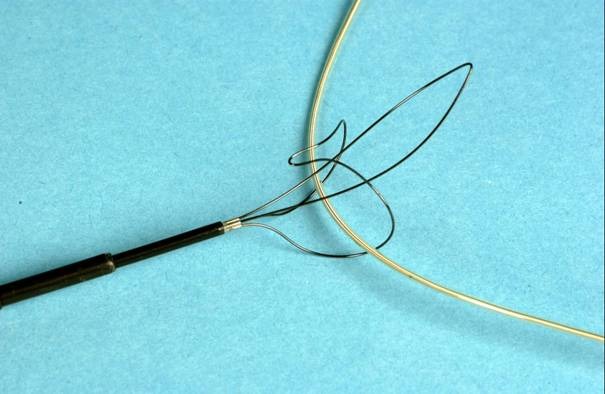
Retriever mechanism of Needle's eye retriever (Cook Inc., In). The lead is first grabbed by the curved hook en secured by advancing the loop through the curved hook

**Figure 4:**
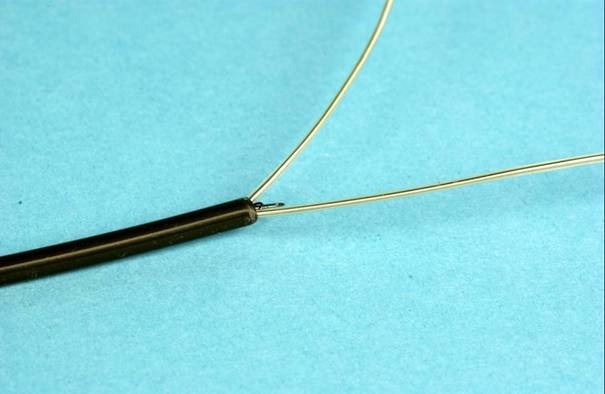
After securing the lead with the retriever it is pulled down whilst the outer sheath is advanced over the doubled up lead

All previous techniques used a superior approach from the lead insertion site, but with the transfemoral approach a long 16 F sheath is introduced via the femoral vein towards the right atrium. Then, a retriever is inserted through the sheath to grab and secure the lead as close to the tip as possible (Figure 3). The lead (with the connector cut off) is pulled inferiorly by the retriever whilst the outer sheath is advanced over the doubled up lead (Figure 4). The proximal part of the lead is pulled down through the fibrous envelope; the distal binding sites are disrupted by the advancing outer sheath. When the sheath reaches the distal electrode, counter traction is again applied. In comparison to the superior approach, the isodiametric lead body is often readily pulled down from the binding sites not hampered by a bulbous tip. Although no locking stylet is used to reinforce the lead, the shorter distance from the retriever to the tip decreases the change of elongating the lead. The technique is especially useful to remove severed and indwelling leads or as back-up after failed superior attempts [22]. Bongiorni et al. first pulled the proximal end of the lead down from the femoral vein, then retrieved the lead via the internal jugular vein.

## Results

The results and complications of different extraction techniques are summarized in Table 1. Although traction and graded traction for lead extraction have been reported on for more than 30 years only Rosenheck et al. recently reported results with the use of rotational forces [14]. Applied on 113 leads in 81 patients this method resulted in complete removal in 86 % of the leads. In 6 % leads dislocated from the myocardium but proximal adhesions impeded removal of the lead. In 8 %, traction was unsuccessful.

Locking stylets have been reported as a stand-alone extraction tool. Alt et al. reported their experience with the VascoExtor stylet (VascoMed GmbH, Weil am Rhein, Germany) in 150 leads in 105 patients, (110 ventricular leads, 40 atrial; passive fixation with tines in 109 leads). Complete removal was possible in 81 % of cases, partial removal in 12 %. Manolis et al. reported success with the same device in 24 out of 25 leads: in 81 % with the sole use of a stylet, in 19 % with additional tools [27]. Results of counter traction technique using conventional sheaths includes also the use of a femoral approach and are reported in the U.S. Lead Extraction Database [20]. Complete removal was achieved in 86.8 % and partial removal in 7.5 % of 2,195 leads. In a paper describing their experience with lead extraction between 1994 and 1996, Byrd et al. reported complete removal in 93 % and partial removal in 5 % of 3540 leads in 2338 patients [21].

Klug et al. reported results of a femoral approach alone with a Needle's Eye snare in 70 leads out of 82 leads extracted in 39 patients [28]. Eighty-seven percent of leads were successfully extracted, 4 % incomplete and 9 % failures.

In a European multi-center study laser sheath extraction was attempted of 179 leads in 149 patients (104 atrial, 57 ventricular, one superior vena cava ICD and 17 ventricular ICD leads) [29]. Complete extraction was achieved in 89.5% of the leads, 6% were partially extracted and 4.5% of the extractions failed. Three out of the 8 failures were completely removed by a femoral non-laser approach, 1 with an alternative superior approach and 1 with thoracotomy.

In a United States registry of 1684 patients undergoing laser sheath extraction of 2561 leads, complete success was achieved in 90 % and partial success in 3 % of leads [30]. In the Excl trial of lead extraction of 287 leads in 166 patients with bipolar electrosurgical sheaths 96% of leads was completely removed, 4 % partially removed and only one lead not removed (laser sheath as an adjunct was used in 2 % of cases) [31].

There is only one trial comparing extraction techniques in a randomized fashion. In the Plexus trial 465 leads were randomized between the laser sheath and conventional sheaths (all investigators had experience with the conventional technique) [23]. Crossover from non-laser to laser was allowed in case of failure of the non-laser approach. Initial attempts at extraction were successful in 94% of leads with the laser sheath against 64 % of leads with the conventional sheaths. However, there was a 33.5 % crossover from conventional techniques to laser. This reflected the greater predictability of laser assisted lead extraction as perceived by the investigators, hence the low threshold for abandoning conventional sheaths if success was not swiftly obtained. If no crossover was allowed one should have expected a success rate in the non-laser group comparable to the published data on conventional extraction [20,21].

Successful lead extraction is especially dependent on implant duration: Byrd et al. reported that the risk of failed or partial doubled for every 3 year of implant [21]. Further, extraction is more likely to be successful with increasing physician's experience, atrial leads and infected leads [32].

## Complications

Using only traction, Rosenheck et al. reported only 1 out of 89 patients with a small pericardial effusion [14]. With conventional sheaths or a femoral workstation, major complications occurred in the U.S. Lead Extraction Database in 2.5 % of the 1,299 patients (hemopericardium or tamponade 1.2 %, hemothorax 0.5 % and pulmonary embolism 0.2 %, death 0.6 %) and in the experience of Byrd et al. in 1.4 % of 2338 patients (thoracotomy in 14 patients, pericardial drainage in 11 patients, transfusions in 4 pts. and 1 death) [20,21].

The use of femoral workstation was accompanied by 2 deaths and transient limb ischaemia in 1 patient [28]. In the 149 patients of the European laser registry, complications included ventricular perforation in one patient; two other perforations were related to the reimplantation of leads and required surgery [29]. There were no fatal complications. In the United States laser registry major complications occurred in 1.9 % of patients, mainly tamponade and haemothorax resulting in the demise of 12 patients (0.8%) [30]. With electrosurgical sheaths, the first results of the Excl trial in 166 patients reported tamponade in 3 patients, haemothorax and AV fistula both in 1 patient.

A direct comparison of complications between techniques is only available from the Plexus trial [23]. Tamponade or hemothorax occurred only 1 in 3 patients randomized to the laser group, one of whom died. Two patients in the laser group and 1 patient in the non-laser had venous thrombotic complications.

It is remarkable that the complication rates between endovascular extraction techniques are comparable. As all different extraction techniques except direct traction rely on countertraction it is not surprising that tamponade resulting from perforation after dislocation of the lead tip is common to all these techniques. A second common predilection site for perforation is the lower superior vena cava. As the vessel wall is sometimes part of the fibrous envelope surrounding the leads, it can be exposed to the mechanical, laser or radiofrequency energy of the sheaths. Electrosurgical sheaths have the possibility of directional application of energy which should avoid the risk of damage to the vessel wall but this has yet to be proven. A femoral approach has an advantage in this respect as the proximal isodiametric part of the lead is most often simply pulled down through the binding sites avoiding applying sheaths in the vena cava superior.

The risk of complications has been associated with physician's experience, number of leads extracted and female sex [20,21,30]. Although implant duration was not linked with complications in any of the cited papers, major complications reported for extraction of Accufix leads (Telectronics Pacing Systems Inc. Englewood, Co, USA) increased from 2 % at one-year implant duration to 8.3 % with an implant duration of more than 5 years as reported by the Accufix Resarch Institute (data available on  www.accufix.com).

## Conclusion

In practice, probably no technique is sufficient by itself to address all lead extractions. The necessity to use tools to extract leads largely depends on the time from implant. Within six months from implant it is rare that traction alone will not suffice. However, from that time on extraction tools are necessary in an increasing number of procedures [32].

Powered sheaths, laser or electrosurgical, provide the best chance of extracting the entire lead, but have a risk of laceration of the veins along the proximal part of the lead. The most versatile technique is probably a femoral workstation and retriever system. It is the only technique suited to remove severed and indwelling leads and is very useful as back-up after a failed or stalled superior venous approach [22].

Although the complication rate of these extraction techniques seems low, their nature is life-threatening and hence extraction is only acceptable for indications that are vital or have a serious morbidity. Ergo, these procedures should be limited to the operation room with adequate cardio-surgical stand-by. The risk of emergency surgery itself is important to consider in advance. In some cases elective surgery is a preferable when risks of endocardial extraction are too high, even in the course of a procedure. There will always be a trade-off between perseverance to obtain successful extraction and complication rate.

All considerations mentioned result that in our opinion only infected pacing systems qualify for primary lead extraction [33]. In high-risk patients or in the absence of systemic infection alternative, albeit less efficient, treatments may be tried before lead extraction is performed. In case of non-functional or superfluous leads there is no proof that abandoning leads has a risk that justifies lead extraction [10] [34]. It is only defendable to extract non-functional leads that have a high chance of success without complications: an implant time less than one year, younger patients and no serious comorbidity.

## Figures and Tables

**Table 1:**
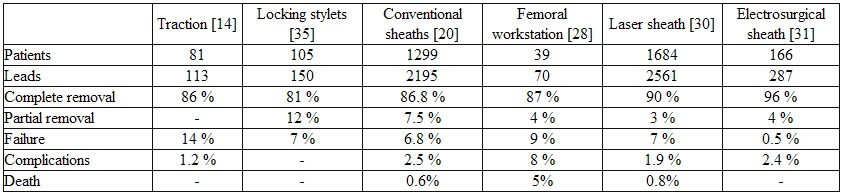
Results and complications of different extraction techniques.
